# EFFECTS OF BARIATRIC SURGERY ON KNEE JOINT PAIN

**DOI:** 10.1590/1413-785220233101e256272

**Published:** 2023-02-20

**Authors:** CLEYTON CHAVES DA ROCHA, RAFAEL SOUZA PINHEIRO, IANN ALAS PAVAN, REGINA YUMI SAITO, PEDRO PEREIRA DA COSTA, HINGRYD EMMYLLY FERREIRA CUNHA

**Affiliations:** 1 Hospital do Servidor Público Municipal de São Paulo, Department of Orthopedics and Traumatology, São Paulo, SP, Brazil.; 2 Hospital do Servidor Público Municipal de São Paulo, Medical Clinic Department, São Paulo, SP, Brazil.

**Keywords:** Pain, Joints, Knee, Obesity, Bariatric Surgery, Dor, Articulações, Joelho, Obesidade, Cirurgia Bariátrica

## Abstract

The World Health Organization (WHO) estimates that by 2025 about 2.3 billion adults will be overweight, with more than 700 million obese. Obese patients with joint pain and reduced physical function represent a challenging group to treat effectively.

**Objective:**

To evaluate patients undergoing bariatric surgery and the implications of this surgery on knee joint pain and to conduct anamnesis and apply specific questionnaires to deepen the discussion and elucidate the knee joint symptoms related to obesity.

**Methods:**

Observational cross-sectional study with tabulation and analysis of collected data.

**Results:**

We obtained a significant result when comparing knee pain pre and post-surgery, in which pain increased by 15.8%.

**Conclusion:**

Although worsening or maintenance of pain may occur, this fact is associated to factors such as the increase of functional activities of a joint that was previously in disuse and the loss of muscle mass as a sustainer. We concluded that the improvement of joint pain complaints were mainly due to the reduction of joint overload. ***Level of Evidence IV, Case Series.***

## RESUMO

A estimativa da Organização Mundial da Saúde é que, em 2025, cerca de 2,3 bilhões de adultos estarão com excesso de peso e, destes, mais de 700 milhões com obesidade. Pacientes obesos com dor nas articulações e função física reduzida representam um grupo desafiador para o tratamento efetivo.

**Objetivo:**

Avaliar pacientes submetidos à cirurgia bariátrica e as implicações dessa cirurgia na dor da articulação do joelho, assim como realizar anamnese e aplicação de questionários específicos para aprofundar a discussão e elucidar os sintomas articulares no joelho relacionados à obesidade.

**Métodos:**

Estudo transversal observacional com tabulação e análise de dados coletados.

**Resultados:**

Obteve-se um resultado significativo na comparação da dor no joelho pré e pós-cirurgia, havendo um aumento da dor de 15,8%.

**Conclusão:**

Ainda que resultados de piora ou manutenção da dor possam ocorrer, associados ao aumento das atividades funcionais de uma articulação até então em desuso e à perda da massa muscular como sustentador, por exemplo, a maioria dos participantes relatou melhora das queixas álgicas articulares, principalmente em decorrência da diminuição da sobrecarga articular. **Nível de Evidência IV, Série de Casos.**

## INTRODUCTION

The World Health Organization (WHO) estimates that by 2025 about 2.3 billion adults will be overweight, with more than 700 million obese.^1^

Obesity affects several systems of the body, including the musculoskeletal system, representing risks that affect the quality of life of affected individuals.^1^ Moreover, mechanical factors with increased load on joints contribute to degenerative joint diseases, significant increase in pain, loss of mobility, and arthropathies.^2^

Obese patients with joint pain and reduced physical function represent a challenging group to treat effectively. Joint pain is associated with osteoarthritis, and obesity is a risk factor for its increased incidence and progression. Other key factors that contribute to joint alterations associated with obesity are advanced age, female gender, smoking, diabetes, and physical workload, especially in the knee.^3^

Bariatric surgery has contributed not only to weight reduction but also to the management of comorbidities associated to obesity.^3^ With weight loss, it is hypothesized that the reduction of overload and the reduction of joint symptoms may be an ally for a more efficient treatment in this group of patients.^4^

This study is justified by the need to deepen, discuss, and elucidate the articular symptoms of the knee, as well as the support of bariatric surgery as a key factor for weight loss and reduction of joint overload. Still by the need to promote new studies related to the casuistry to help in the reflection of such a relevant theme for public health.

### Primary objective

To evaluate patients undergoing bariatric surgery and the implications of this surgery on knee joint pain.

### Secondary objective

To perform anamnesis, apply specific questionnaires to deepen the discussion and elucidation of knee joint symptoms that are related to obesity.

## MATERIALS AND METHODS

The method defined for the study is cross-sectional, observational with tabulation and analysis of data collected throughout the study.

For data collection in the study, a questionnaire was used to evaluate the profile of the patients regarding gender, comorbidities, body mass index (BMI), consultation in the orthopedic clinic, physical activity, and use of medications or complementary therapies for pain control.

A modified Nordic Musculoskeletal Symptoms Structured Questionnaire (QNSO) was used to evaluate knee pain. The instrument consists of multiple or binary choices as to the occurrence of symptoms in the various anatomical regions in which they are most common. The respondent should report the occurrence of symptoms considering the 12 months and seven routine days in the year preceding the interview, as well as report the occurrence of absence from activities.^5^ ^),(^ ^6^

The osteoarthrosis-specific quality of life questionnaire (WOMAC) was also used. This questionnaire has been widely used in research and was validated in 2002 for use in the Brazilian population, maintaining its original parameters. It assesses three domains: pain, stiffness, and function, considered central to the evaluation of patient outcomes and the gold standard of measurement in knee osteoarthritis. After the answers, they are transformed into a score based on a Likert-type scale ranging from 0 to 4 points. The highest score reached by the sum of the points shows greater impairment in the domains cited.^7^

We evaluated patients seen at the Orthopedics and Traumatology Department of the Hospital do Servidor Público Municipal of São Paulo who underwent bariatric surgery. They met the criteria of BMI of 40 kg/m^2^ or BMI of 35 kg/m^2^ to 40 kg/m^2^ with comorbidities, BMI of 30 kg/m^2^ to 35 kg/m^2^ in Diabetes Mellitus II refractory to clinical treatment.^8^

All patients signed an informed consent form. The protocol was approved by the Ethics Committee of Hospital do Servidor Público Municipal of São Paulo under opinion number 4.589.682.

Inclusion criteria:

- Patients under outpatient follow-up;
- Clinical diagnosis of obesity subjected to bariatric surgery.

Exclusion criteria:

- Cognitive alterations that prevent the application of the questionnaire;
- Loss of outpatient follow-up;
- Bariatric patients under 18 years.

## RESULTS

In the analyzed period, 38 people who underwent bariatric surgery answered the questionnaires. According to the analyses, 32 (84.2%) of the 38 research participants (84.2%) were female and six (15.8) were male.

When analyzing the BMI, we observed a higher prevalence of overweight people (BMI 25 to 29.9) and grade I obesity (BMI 30-34.9); characterized by 12 (32%) and 15 (39%) respectively.

When we asked the participants if they had ever been to an orthopedic consultation, 20 people (52.6%) answered that they had never been to the specialty, seven people (18.4%) had been before and after bariatric surgery, four people (10.5%) went after bariatric surgery, and seven people (18.4%) went only before bariatric surgery.

Of the people who visited the orthopedic specialist, nine people (23.7%) reported pain or discomfort in the knee (Table 1).

**Table 1:** Knee-related pain after orthopedic consultation.

KNEE_PAIN_CLASS				
	Frequency	%	% Valid	% Cumul.
**No**	10	26.3	26.3	26.3
**Yes**	9	23.7	23.7	50.0
**Not applicable**	19	50.0	50.0	100.0
**Total**	38	100.0	100.0	

%: percentage; % Cumul.: cumulative percentage.

All patients (100%) underwent surgery using the Roux-en-Y gastroplasty technique (gastric bypass).

Of the 38 respondents, 33 (86.8%) did not perform physical exercises before bariatric surgery. Of the five people who reported doing some type of physical activity, two people (94%) walked, one person (2%) danced, one person (2%) did water aerobics, and one person (2%) did weight-training and dance classes. Regarding the weekly frequency, three people (7.9%) did their activities three times per week.

When comparing the performance of physical activities before and after bariatric surgery, we observed a considerable increase of people who started to perform physical activities after the surgery. The number of respondents who started performing physical activities after surgery increased from five (13.2%) to 27 (71.1%). Walking (7; 18.4%) and water aerobics (2; 5.3%) were the most adhered activities. Some also associated two activities, such as hydrogymnastics and weight-training (2; 5.3%). Others performed only weight-training (2; 5.3%).

Regarding the number of times per week, we noticed a significant increase. Most individuals (8; 21.1%) started doing physical activity three to five times per week. Two people (5.3%) started doing activity six times per week, and one person (2.6%) every day of the week.

In the postoperative evaluation with the QNSO, 22 patients (57.9%) reported knee pain that limited their daily activities (Table 2). On the pain scale, from 0 to 10, the reported average was 5.39.

**Table 2:** Responses to the modified Nordic Musculoskeletal Symptoms Structured Questionnaire regarding the knee.

KNEE_PAIN	Frequency	%	% Valid	% Cumul.
**No**	15	39.5	40.5	40.5
**Yes**	22	57.9	59.5	100
**Total**	37	97.4	100	
**No data**	1	2.6		
**Total**	38	100		

%: percentage; % Cumul.: cumulative percentage.

The patients were analyzed in relation to knee pain by the WOMAC. On average, 50% of the interviewees reported feeling no pain during the activities to evaluate knee pain after bariatric surgery. Regarding knee pain after light walking on flat ground, 20 people (52.6%) reported feeling no pain and mild pain in 36.8% of the cases. Only one person (2.6%) reported a great difficulty when walking on flat ground.

About pain when climbing or descending stairs, the patients reported no pain (12; 31.6%), mild pain (14; 36.8%), and moderate pain (9; 23.7%). Analyzing the difficulty when going downstairs, 14 people (36.8%) reported mild difficulty or no difficulty (36.8%). We found no difference regarding climbing stairs, as 14 people (36.8%) reported slight difficulty or no difficulty (34.2%).

Regarding pain when lying down at night, 19 people (50%) reported feeling no pain and 16 (42.1%) reported mild pain. A total of 42.1% reported feeling no pain sitting or standing up and 39.5% reported feeling mild pain. Only three people reported feeling moderate (15.8%) or severe (7.9%) pain when standing up. Regarding stiffness when waking, lying down, sitting and/or resting, nine people (23.7%) had moderate or strong stiffness (5.3%).

Only three people (7.9%) reported strong difficulty when getting up from a chair and eight people (21.1%) reported moderate difficulty. Most patients had no (44.7%) or mild (42.1%) difficulty in standing up after 72 h from surgery.

Most patients (18; 47.4%) had no difficulty getting out of bed, lying in bed (22; 57.9%), putting on the stocking (15; 39.5%), and taking off the stocking (16; 42.1%).

Regarding daily activities, more than half (55.3%) had no difficulty getting in or out of the shower; 15 people (39.5%) had mild or no difficulty (39.5%) in sitting down or getting up from the toilet.

When questioned about heavy household tasks, the number of people who reported no difficulty or moderate difficulty was the same, 12 people, representing 31.6% for each level. For light household tasks, one person (2.6%) reported strong difficulty.

Comparing the data compiled in the initial questionnaire that outlined the profile of the research participants and the QNSO, we had a significant result regarding the knee pain pre and post surgery. There was a 15.8% increase in pain (Table 3).

**Table 3:** Cross between knee pain reported at initial visit to orthopedics (pre bariatric surgery) and after response to questionnaires (post bariatric surgery).

KNEE_PAIN_PRE × KNEE_PAIN_POST crossing				
		Post knee pain		Total
**Pre knee pain**		No	Yes	
	No	36.8%	15.8%	52.6%
	Yes		47.4%	47.4%
**Total**		36.8%	63.2%	100%
Fisher’s test: p = 0.002				

## DISCUSSION

With the rising levels of obesity in Brazil, the risk of osteoarthritis increases. Obesity is one of the risk factors for osteoarthritis that can be modified, thus maintaining adequate body weight at all ages is recommended to avoid complications.^9^ A variety of methods can be used to treat osteoarthritis, including medications, exercise (with or without diet), and bariatric surgery.

In the United States of America (USA), the overall prevalence of knee pain in the adult population is 20%, with more than 61 million people affected. By 2025, it is projected to increase to 25% of the affected population.^3^

Studies show that for every kilogram lost, the load on the knee from excess weight is reduced twice. With this comorbidity increasingly present in young patients, the demand for “faster” weight loss has increased dramatically, and thus bariatric surgery is widespread.^3^

Vicent et al.^4^ found data suggesting that individuals with knee joint pain complaints in the preoperative period of bariatric surgery had improvements, and it was considered a predictor of a better quality of life.

According to Abu-Abeid et al.^10^ the female population (68%) was the most affected by obesity, whereas in the study by Groen et al.,^11^ it corresponded to (72%) of people with obesity. These studies corroborate the results of our study, in which the female population (84%) composed most cases. The prevalence of obesity in the female population increases in the age group starting at 54 years, and our average among women is 48.5 years.^12^

Patients subjected to weight loss present gait alterations, such as increased flexion and extension range of motion, external and internal rotation, and better distribution of joint stress. Improvements in gait kinematics, stride length, and cadence are also observed. This contributed to the results found, such as an increase in physical activity after bariatric surgery, considering the facilitation of patient mobility.^13^

Several authors correlate knee pain and obesity due to the overload that this joint suffers with overweight.^3^ ^),(^ ^11^ ^),(^ ^14^ ^),(^ ^15^ Growing evidence indicates that regardless of the method of weight loss, reducing body fat can reduce the mechanical and biochemical stressors that contribute to joint degeneration. Compared with our survey, although all patients had bariatric surgery, there was a significant increase in exercise post surgery.^16^ Before bariatric surgery, five people (13.2%) did some type of physical activity. After the surgery, this number increased to 27 (71.1%).

According to the study by Vincent et al.,^15^ the improvement in lower back and knee pain after three months of bariatric surgery was significant. Those who reported no pain went from 25% to 50% in the third month after the surgery. The most obvious pain reduction effects occurred in the knee and lower back compared to other joints. Weight loss seemed to be directly related to the magnitude of pain relief in the lower back and knee, but not as strongly related for other joints. In our study, several joints were evaluated through the questionnaires. The biggest complaints of the patients are lower back pain (60.5%), knee pain (57.9%), and shoulder pain (44.7%), corroborating the studies.

When we compared our survey with other studies in comparison to the WOMAC Questionnaire, we noticed that the complaint of pain is mostly none (36.8%) or mild (47.4%) if evaluated after bariatric surgery.^17^

Regarding stiffness, we found most patients complained of none (44.7%) or mild stiffness (23.7%). Patients complained of no (42.1%) or mild (34.2%) degree of difficulty in function, which may be correlated with the significant increase in patients who started exercises after bariatric surgery. The performance of constant exercises as an aid in improving functional capacity and reducing joint stiffness is cited in several studies.^3^ ^),(^ ^17^ ^),(^ ^18^

Regarding the data collected with the QNSO, we verify that 57.9% complained of knee pain after bariatric surgery. However, when we compared the knee pain reported in the pre-surgery questionnaire, we observed an increase in pain of 15.8%.

Bariatric surgery with the Roux-en-Y gastric bypass technique triggers considerable loss of lean body mass, a reflection of the inadequate energetic-protein supply, which may cause a disorder in metabolism, increased proteolysis due to major restrictions to provide substrate to gluconeogenesis. This tends to worsen joint pain, corroborating the results found of increased joint pain in 15.8% of patients in the postoperative period.^19^

Hamdi et al.^3^ reinforces that there is a need for personalized exercise programs for bariatric surgery patients to strengthen their muscles, preserve their lean mass and thus prevent the progression of knee pain due to overexertion.

## CONCLUSION

This research assumed that weight loss and the reduction of joint overload would contribute to the reduction of joint pain complaints. During the study, we found that female subjects constituted the largest number of participants in the research. After the surgery, more people started to perform physical activities and the frequency of regular physical exercises increased.

There was also considerable or total improvement in pain complaints, stiffness, and functionality when the WOMAC questionnaire was analyzed. When the QNSO and the patient profile questionnaire were analyzed, we observed the maintenance or non-expressive but significant increase in the pain complaint of the knee.

We concluded that the improvement of joint pain complaints were mainly due to the reduction of joint overload. Even though worsening or maintenance of pain may occur, this fact is associated to factors such as the increase of functional activities of a joint that was previously in disuse and the loss of muscle mass as a sustainer.
